# A blinded comparison of fluticasone propionate with budesonide via powder devices in adult patients with moderate-to-severe asthma: a clinical evaluation

**DOI:** 10.1155/S0962935196000555

**Published:** 1996-10

**Authors:** N. Ringdal, P. Swinburn, R. Backman, P. Plaschke, A. P. Sips, P. Kjaersgaard, G. Bratten, T. A. J. Harris

**Affiliations:** 1 Molde County Hospital Norway; 2 Mercy Specialist Centre New Zealand; 3 Meltola Hospital Finland; 4 Sahlgren's Hospital Sweden; 5 The Polikliniek Kanalweg The Netherlands; 6 Glaxo Wellcome AS Norway; 7 International Medical Affairs Glaxo Wellcome UK

## Abstract

*In Vitro* and *in vivo* data have demonstrated that there are detectable differences between inhaled corticosteroids commonly used to treat asthma. However, controversy still remains as to whether these differences translate into clinical benefits. This 12-week, international, randomized, doubleblind, parallel-group study was undertaken to compare the efficacy and safety of fluticasone propionate (FP) 800 μg daily, administered as a powder via the Diskhaler^®^, and budesonide (BUD) 1600 μg daily, administered using the Turbuhaler^®^, in adult patients with moderate-tosevere asthma. A total of 518 patients participated in the study, 256 of whom received FP and 262 BUD. Assessment of mean morning peak expiratory flow (PEF) over the 12-week treatment period revealed a statistically significant difference in efficacy between FP 800 μg daily and BUD 1600 μg daily in favour of FP (*p* = 0.003), with an overall improvement of 20.9 l/min with FP compared with 12.4 l/min on BUD. Statistically significant differences in favour of FP were seen over the 12 weeks for mean evening PEF (*p* = 0.04), diurnal PEF variation (*p* = 0.03) and percentage predicted PEF (*p* = 0.003), as well as forced expiratory volume (*p* = 0.008), forced vital capacity (*p* = 0.02) and PEF (*p* = 0.005) measured at clinic visits. The median percentage of symptom-free nights increased over the 12-week study period in both treatment groups, with similar changes seen for the median percentage of days with symptom score < 2, rescue medication use and exacerbations of asthma. The incidence of adverse events was found to be comparable in the two treatment groups. The geometric mean ratios of serum cortisol levels were found to be 1.03 for FP, indicating no mean hypothalamic-pituitary-adrenal axis suppression from baseline, and 0.93 for BUD (*p* = 0.0002 compared with FP). In summary, FP 800 μg daily showed a greater efficacy/safety ratio in the treatment of moderate-to-severe asthma than BUD 1600 μg daily.


Mediators of Inflammation, 5, 382–389 (1996)

### A blinded comparison of fluticasone propionate with budesonide via powder devices in adult patients with moderate-to-severe asthma: a clinical evaluation

N. Ringdal,[1] P. Swinburn,[2] R. Backman,[3] P. Plaschke,[4] A. P. Sips,[5] P. Kjaersgaard,[6] G. Bratten[6] and T. A. J. Harris[7,CA]

[1]Molde County Hospital, Norway; [2]Mercy Specialist Centre, New Zealand; [3]Meltola Hospital, Finland; [4]Sahlgren's Hospital, Sweden; [5]The Polikliniek Kanalweg, The Netherlands; [6]Glaxo Wellcome AS, Norway; and [7]International Medical Affairs, Glaxo Wellcome, UK

[CA] Corresponding Author
Tel: (+44) (0)181 990 8467
Fax: (+44) (0)181 990 4326

**IN VITRO and *in vivo* data have demonstrated that there are detectable differences between inhaled corticosteroids commonly used to treat asthma. However, controversy still remains as to whether these differences translate into clinical benefits. This 12-week, international, randomized, double-blind, parallel-group study was undertaken to compare the efficacy and safety of fluticasone propionate (FP) 800 μg daily, administered as a powder via the Diskhaler®, and budesonide (BUD) 1 600 μg daily, administered using the Turbuhaler®, in adult patients with moderate-to-severe asthma. A total of 518 patients participated in the study, 256 of whom received FP and 262 BUD. Assessment of mean morning peak expiratory flow (PEF) over the 12-week treatment period revealed a statistically significant difference in efficacy between FP 800 μg daily and BUD 1 600 μg daily in favour of FP ($p = 0.003$), with an overall improvement of 20.9 l/min with FP compared with 12.4 l/min on BUD. Statistically significant differences in favour of FP were seen over the 12 weeks for mean evening PEF ($p = 0.04$), diurnal PEF variation ($p = 0.03$) and percentage predicted PEF ($p = 0.003$), as well as forced expiratory volume ($p = 0.008$), forced vital capacity ($p = 0.02$) and PEF ($p = 0.005$) measured at clinic visits. The median percentage of symptom-free nights increased over the 12-week study period in both treatment groups, with similar changes seen for the median percentage of days with symptom score < 2, rescue medication use and exacerbations of asthma. The incidence of adverse events was found to be comparable in the two treatment groups. The geometric mean ratios of serum cortisol levels were found to be 1.03 for FP, indicating no mean hypothalamic-pituitary-adrenal axis suppression from baseline, and 0.93 for BUD ($p = 0.0002$ compared with FP). In summary, FP 800 μg daily showed a greater efficacy/safety ratio in the treatment of moderate-to-severe asthma than BUD 1 600 μg daily.**

**Key words:** Asthma, Budesonide, Double-blind comparison, Fluticasone propionate

### Introduction

The objectives of anti-asthma therapy are to abolish symptoms, prevent exacerbations and maintain normal lung function. A number of therapeutic approaches are available for the clinical management of this condition, including bronchodilators and anti-inflammatory agents. In general, a stepwise approach is recommended. Minimum therapy to maintain effective control should be used, with the level of therapy increased with increasing asthma severity.

Inhaled anti-inflammatory drugs are now recommended in international guidelines for the treatment of even mild asthma, with corticosteroids being the therapy of choice.[1,2] The introduction of inhaled corticosteroids in the 1970s represented a significant advance in the treatment of asthma, combining high topical potency with low systemic activity compared with oral administration. Although adequate asthma control can be achieved with low doses of inhaled corticosteroids (200–800 μg daily), some patients may continue to have reduced lung function and experience asthma symptoms necessitating the administration of higher doses

© 1996 Rapid Science Publishers

(1 600–2 000 μg daily).[1] Preliminary data suggest that high doses of inhaled corticosteroids may have a role in the treatment of asthma exacerbations.[3] However, while older corticosteroids such as budesomide (BUD) and beclomethasone dipropionate (BDP) generally do not produce clinically significant systemic adverse effects at low doses, they may suppress the hypothalamic-pituitary-adrenal (HPA) axis function at higher doses.[4–6] There is therefore a clear need for new inhaled corticosteroids with a high topical potency combined with a reduced potential for systemic effects.

Fluticasone propionate (FP) is a topical corticosteroid which has been shown *in vitro* (Table 1) to be more potent than either BDP or BUD, with the advantage of a lower oral bioavailability.[7–11] This is significant, as up to 80% of any inhaled dose may be swallowed and possibly absorbed into the circulation, increasing the potential for systemic adverse effects.[4] However, there has been some criticism as to the relevance of *in vitro* data being used to predict improved clinical benefit when treating patients with asthma. Clinical experience to date has shown that at equal doses, FP is more effective than either BDP or BUD for the treatment of moderate-to-severe asthma in adults[12,13] and to have an approximate 2:1 potency ratio compared with other inhaled corticosteroids.[14–17]

This double-blind study was designed to show equivalent efficacy and compare the tolerability of FP at a relatively high dose of 800 μg daily administered as a powder in adults with moderate-to-severe asthma, versus BUD 1 600 μg daily, an established treatment in this indication.

### Patients and Methods

### Study design

This was an international, double-blind, double-dummy, parallel-group study, with a treatment duration of 12 weeks preceded by a 2-week run-in period. Study visits took place at the start of the run-in and treatment periods, and after 4, 8 and 12 weeks of therapy. Patients were randomized to receive either FP 800 μg daily, administered as a powder via the Diskhaler®, or BUD 1 600 μg daily, administered by the Turbuhaler®. Patients received their usual inhaled steroid during the run-in period and switched to the study drug at the start of the treatment period.

Salbutamol was used throughout the study as rescue medication. All concomitant asthma medication (except oral corticosteroids and short-acting $\beta_2$ agonists other than salbutamol) were permitted, provided they had been taken at a constant dosage for 4 weeks prior to visit 1 and during run-in. Any changes in concomitant therapy were documented.

The study was conducted to Good Clinical Practice in accordance with the Declaration of Helsinki (Hong Kong Amendment 1989), and approved by local ethics committees. Patients gave written or witnessed oral consent to participate.

### Patients

Patients were aged 18 to 75 years, with a documented clinical history of reversible airways obstruction treated with inhaled steroids at a constant dosage for 4 weeks prior to study entry (BDP or BUD at 800–1 600 μg/day or FP at 400–800 μg/day). All patients were required to have: (i) a forced expiratory volume in 1 s ($FEV_1$) of between 45% and 90% of the predicted value; (ii) a clear response to bronchodilator therapy, defined as a mean morning peak expiratory flow (PEF) over the last 7 days of the run-in period of $\leqslant$ 90% of the response obtained following administration of salbutamol 400 μg or 800 μg at the start of the treatment period; (iii) required two or more doses of a bronchodilator, or to have had asthma symptoms (total score of $\geqslant$ 2) on at least four of the last 7 days of the run-in period.

Patients were excluded from the study if their reversible airways obstruction was unstable; if they had received oral corticosteroids; had a

**Table 1.** In vitro potency measures

| Compound | CD4+ T-cells: cytokine secretion ($IC_{50}$ [nM] for $IL_5$)[9] | Histamine release $IC_{50}$ (M)[11] | Eosinophil survival ($IC_{50}$[nM])[10] |
|---|---|---|---|
| Beclomethasone dipropionate (BDP) | 7.7 ± 1.9 | $1.0 \times 10^{-9}$ | 138.7 |
| Triamcinolone acetonide | 9.8 ± 5.1 | $2.0 \times 10^{-8}$ | 23.8 |
| Budesonide (BUD) | 1.7 ± 0.7 | $5.9 \times 10^{-10}$ | 8.5 |
| Mometasone furoate | 0.3 ± 0.1 | $3.0 \times 10^{-10}$ | NA |
| Fluticasone propionate (FP) | 0.2 ± 0.1 | $3.0 \times 10^{-11}$ | 1.7 |

respiratory tract infection or been admitted to hospital for respiratory disease during the 4 weeks prior to study entry; or if they had required 16 or more doses of rescue salbutamol during the last 6 days of the run-in period. Patients with concomitant disease which might have interfered with assessment of study medication, hypersensitivity to inhaled corticosteroids, evidence of alcohol or drug abuse, and pregnant or lactating women were also excluded from participation.

### Efficacy assessments

The primary efficacy variable was morning PEF, measured every day at weeks 1–12, with secondary variables of evening PEF, day- and night-time symptom severity, symptomatic bronchodilator use, clinical lung function measurements (PEF, $FEV_1$ and forced vital capacity (FVC)) and exacerbation rate. Patients measured their own PEF in the morning and evening, using a mini-Wright peak flow meter. PEF was measured in the morning before taking any medication, and in the evening at least 4 h after bronchodilator use (preferably at least 12 h after taking a long-acting or oral $\beta_2$ agonist, or theophylline). Three measurements were taken, and the highest one recorded in a daily diary record card.

Symptom severity and bronchodilator use were recorded by the patients each day on a diary card. Day-time symptoms were rated on a scale of 0 to 5 as follows: 0 = no symptoms; 1 = symptoms for one short period; 2 = symptoms for two or more short periods; 3 = symptoms for most of the day which did not affect daily activities; 4 = symptoms for most of the day which did affect daily activities; 5 = symptoms so severe the patient could not work or perform normal daily activities. Night-time symptoms were rated on a scale of 0 to 4: 0 = no symptoms; 1 = symptoms causing the patient to wake once or early; 2 = symptoms causing the patient to wake twice or more (including early waking); 3 = symptoms causing the patient to be awake most of the night; 4 = symptoms so severe the patient did not sleep at all. Exacerbation of asthma was defined as requiring salbutamol more than eight times per day on more than 3 days during any 6-day period, or a PEF value of < 85% of the baseline morning value on 3 days during any 6-day period.

Lung function measurements were performed at the clinic at each of the five study visits, and the highest of three PEF, $FEV_1$ and FVC values were recorded. Patients were required to have withheld use of a short-acting bronchodilator for 4 h, and a long-acting bronchodilator or theophylline for 12 h before attending the clinic. All adverse events were recorded, and serum cortisol measurements were taken at baseline and at the end of the study.

### Statistical analysis

For calculation of sample size, treatment groups were considered equivalent if the 95% confidence interval (CI) for the difference between treatments was ≤ 15 l/min. Assuming a standard deviation of 30–45 l/min, as seen in previous studies, 260 evaluable patients per treatment group were required to ensure a power of at least 80%. It was anticipated that a maximum number of 700 patients, recruited from approximately 50–60 centres, would be required to achieve this figure.

Data from the daily diary cards completed during the run-in period were used to establish baseline values. For the assessment period, data were analysed for weeks 1–4, 5–8, 9–12 and 1–12. All efficacy data were analysed on an intent-to-treat basis. To be included in the analysis of a variable, patients were required to have provided data from at least 1 day of the last week of the run-in period and at least 1 day of the assessment period.

Diary card data from the treatment period were used to calculate mean morning and evening PEF, the diurnal variation in PEF (defined as the mean difference between the previous evening and next morning values) and percentage predicted PEF for both treatments over each assessment period. An analysis of covariance was performed on these variables using baseline values as a covariate.

In addition, the following variables were analysed by treatment and assessment period: percentage of days with a symptom score < 2; percentage of symptom-free nights; median night-time symptom score; median day-time symptom score; percentage of days and nights when additional bronchodilator medication was not required; median day- and night-time rescue medication requirement. These variables were analysed by the Wilcoxon rank-sum test. An analysis of covariance was performed on the measurements of PEF, $FEV_1$ and FVC taken in the clinic, using baseline values as a covariate.

All patients randomized to treatment were included in the safety analysis. Differences in the number of exacerbations of asthma, withdrawals and adverse events between treatment groups were compared using the chi-squared test. Serum cortisol data were log transformed prior to analysis of covariance, using baseline values as a covariate. All statistical tests performed were two-sided, with $p$-values of $< 0.05$ considered significant. No adjustment to the $p$-values were performed to take into account multiple significance testing.

### Results

### Demography

Of the 518 patients randomized to treatment, 256 received FP and 262 BUD. Patient characteristics are summarized in Table 2. Overall, there was a slightly higher proportion of males compared to females entering the study (53.9% *vs* 46.1%). This difference was more marked in the FP group (57.4% *vs* 42.6%) compared with the BUD group, where the proportions were equal (50.4% *vs* 49.6%). Apart from this finding, the two treatment groups were found to be well matched for all key demographic variables at baseline.

A total of 49 patients withdrew from the study; 25 from the FP group and 24 from the BUD group. Reasons for withdrawal included adverse events (ten on FP *vs* 13 on BUD), lack of efficacy (two FP, one BUD), non-compliance (three FP, two BUD), failure to return (four FP, three BUD), not fulfilling entry criteria (four FP, three BUD) and other reasons (two FP, two BUD), with no significant differences between treatment groups.

**Table 2.** Patient characteristics at baseline

| Demographic variable | FP 800 µg | BUD 1 600 µg |
|---|---|---|
| Patients *n* | 256 | 262 |
| Sex | | |
| Male *n* (%) | 147 (57.4) | 132 (50.4) |
| Female *n* (%) | 109 (42.6) | 130 (49.6) |
| Age (mean [SD] years) | 47.6 (14.8) | 48.3 (14.0) |
| Caucasian *n* (%) | 227 (88.7) | 238 (90.8) |
| Smokers *n* (%) | 43 (16.8) | 54 (20.6) |
| Duration of RAO [mean (SD) years] | 17.4 (14.6) | 17.7 (12.8) |
| No. exacerbations requiring change of medication in last 12 months (mean [SD]) | 1.1 (1.5) | 1.1 (2.3) |
| Patients hospitalized at least once in last 12 months due to exacerbation *n* (%) | 27 (10.5) | 29 (11.1) |
| Inhaled corticosteroid treatment (µg/day) *n* (%) | | |
| BDP 400–< 1 200 | 62 (24.2) | 61 (23.3) |
| BDP 1 200–2 000 | 12 (4.7) | 27 (10.3) |
| BUD 400–< 1 200 | 95 (37.1) | 93 (35.5) |
| BUD 1 200–2 400 | 57 (22.3) | 48 (18.0) |
| FP 400–500 | 24 (9.4) | 26 (9.9) |
| FP 501–1 000 | 6 (2.3) | 7 (2.3) |

BDP = beclomethasone dipropionate; BUD = budesonide; FP = fluticasone propionate; RAO = reversible airways obstruction.

### Efficacy

Morning and evening PEF improved over baseline values in both treatment groups during the course of the trial. For morning PEF, greater improvements were seen on FP than on BUD (Fig. 1), resulting in statistically significant differences favouring FP at each assessment interval ($p = 0.0007$, weeks 1–4 and 5–8; $p = 0.002$ weeks 9–12) and over the whole 12-week treatment period ($p = 0.003$, Table 3). The overall improvement in mean morning PEF (baseline *vs* weeks 1–12) was 20.9 l/min (95% CI: 16.2–25.5) with FP compared with 12.4 l/min (95% CI: 8.1–16.7) on BUD. Morning PEF improved by ≥ 10% over the whole treatment period in 27% of patients on FP compared with 18% on BUD.

Similar results were seen for evening PEF (Fig. 2, Table 3), with 20% of patients taking FP improving by ≥ 10% over the whole treatment period compared with 15% on BUD.

Analysis of the diurnal variation in PEF and percentage predicted PEF, as well as clinic assessments of lung function (PEF, $FEV_1$ and FVC) also showed improvement in both treatment groups, with intergroup differences for all parameters evaluated attaining statistical significance in favour of FP over the 12-week treatment period (Table 3).

Patient diary card data revealed an improvement in symptoms experienced by the patients over the course of the trial, with comparable stability in median day- and night-time symptom scores in both treatment groups. At baseline, patients in both treatment groups had a symptom score $< 2$ on only approximately 30% of days. Between weeks 9 and 12, however, this had increased to 95% with FP and 89% with BUD (Table 4). Similarly, the percentage of symptom-free nights improved from 28% at baseline to 80% between weeks 9 and 12 for FP, and 33% at baseline to 85% between weeks 9 and 12 for BUD (Table 4). There were no statistically significant differences between groups after controlling for centre. For all measures of symptom severity, the largest improvement occurred during the first 4 weeks of treatment in both treatment groups.

The improvement in asthma symptoms of patients receiving therapy was also reflected in a decrease in patients' additional day- and night-time bronchodilator use. For the percentage of days with no additional bronchodilator use, there was a statistically significant difference in favour of FP over the period 9–12 weeks (Table 5). For the percentage of nights with no additional bronchodilator use, similar improve-

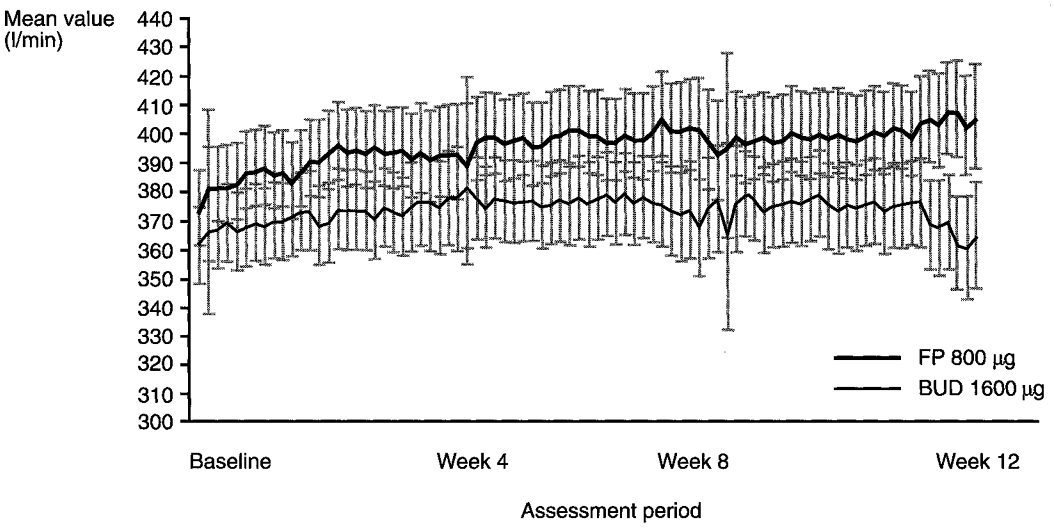

FIG. 1. Morning peak expiratory flow recorded from patient diary record cards.

**Table 3.** Changes in lung function on therapy

| | FP 800 μg | BUD 1 600 μg | *p* value |
|---|---|---|---|
| Mean (SD) morning PEF (l/min) | | | |
| Baseline | 372.8 (102.8) | 361.4 (105.5) | NS |
| 1–12 weeks | 393.9 (105.3) | 372.6 (107.6) | 0.003 |
| Mean (SD) evening PEF (l/min) | | | |
| Baseline | 390.3 (103.6) | 379.6 (103.3) | NS |
| 1–12 weeks | 404.1 (103.9) | 386.4 (106.1) | 0.04 |
| Mean (SD) diurnal variation (l/min) | | | |
| Baseline | 17.4 (31.9) | 17.9 (31.0) | NS |
| 1–12 weeks | 10.1 (23.5) | 13.7 (25.1) | 0.03 |
| Mean (SD) percentage predicted PEF (l/min) | | | |
| Baseline | 79.5% (17.9) | 78.2% (17.6) | NS |
| 1–12 weeks | 84.1% (19.3) | 80.7% (18.0) | 0.003 |
| Mean (SD) clinic PEF (l/min) | | | |
| Baseline | 401.3 (99.7) | 385.0 (109.5) | NS |
| Week 12 | 426.1 (110.8) | 405.9 (109.0) | 0.005 |
| Mean (SD) clinic $FEV_1$ (l) | | | |
| Baseline | 2.26 (0.69) | 2.21 (0.73) | NS |
| Week 12 | 2.38 (0.77) | 2.27 (0.77) | 0.008 |
| Mean (SD) clinic FVC (l) | | | |
| Baseline | 3.46 (0.97) | 3.35 (1.02) | NS |
| Week 12 | 3.53 (0.99) | 3.37 (1.01) | 0.02 |

BUD = budesonide; $FEV_1$ = forced expiratory volume in 1 s; FP = fluticasone propionate; FVC = forced vital capacity; PEF = peak expiratory flow.

ments were seen over the course of the trial in both patient groups; this decrease was most marked during the first 4 weeks of treatment (Table 5).

There were no significant differences in the total number of patients reporting exacerbations of asthma (as defined in the protocol) between the two groups. In all, 41 (16.0%) patients on FP and 51 (19.5%) of those who received BUD experienced exacerbations of asthma during the course of the trial.

### Safety

Adverse events occurred with a similar frequency in both patient groups, with 158 (61.7%) patients treated with FP and 161 (61.5%) of those who received BUD reporting an adverse event during the course of the trial. Seven (2.7%) patients in the FP group and 9 (3.4%) on BUD reported adverse events as serious. However, in only one patient in the BUD group were these considered to be possi-

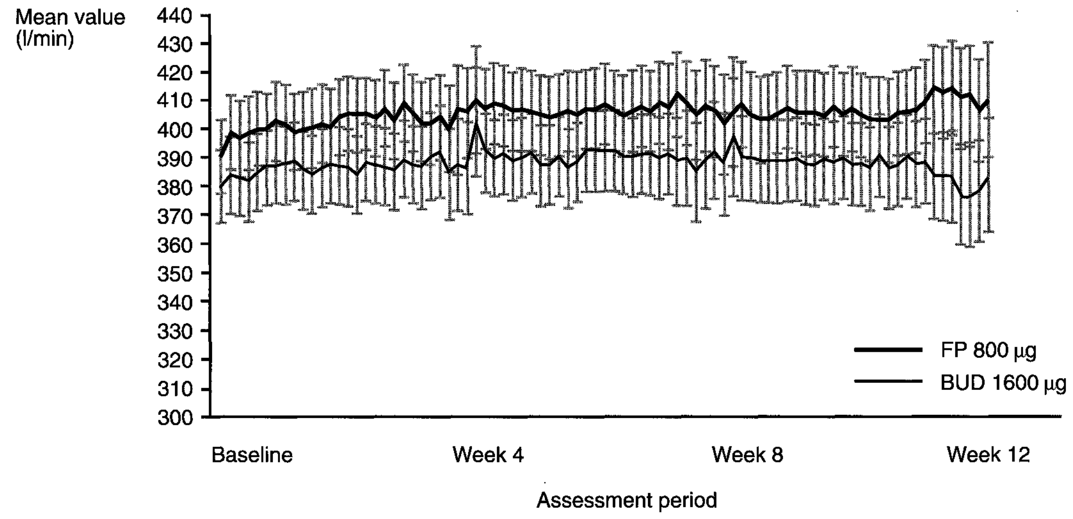

FIG. 2. Evening peak expiratory flow recorded from patient diary record cards.

**Table 4.** Day- and night-time symptom severity from the patient diary cards

| | Percentage of days with symptom score < 2 (median) | | | Percentage of symptom-free nights (median) | | |
|---|---|---|---|---|---|---|
| | FP 800 μg | BUD 1 600 μg | *p* value | FP 800 μg | BUD 1 600 μg | *p* value |
| Baseline | 33.3 | 33.3 | – | 28.6 | 33.3 | – |
| Week 1–4 | 82.5 | 81.8 | 0.17 | 73.0 | 73.5 | 0.18 |
| Week 5–8 | 89.5 | 86.4 | 0.16 | 77.8 | 81.5 | 0.15 |
| Week 9–12 | 95.2 | 89.3 | 0.50 | 80.0 | 85.3 | 0.37 |
| Week 1–12 | 85.7 | 83.3 | 0.42 | 73.2 | 77.5 | 0.43 |

The range of minimum and maximum values for percentage of symptom-free nights at baseline was 0–88%. All other ranges were 0–100%. BUD = budesonide; FP = fluticasone propionate.

**Table 5.** Additional bronchodilator use recorded by the patients

| | Percentage of days with no additional bronchodilator use (median) | | | Percentage of nights with no additional bronchodilator use (median) | | |
|---|---|---|---|---|---|---|
| | FP 800 μg | BUD 1 600 μg | *p* value | FP 800 μg | BUD 1 600 μg | *p* value |
| Baseline | 0.0 | 0.0 | – | 26.7 | 28.6 | – |
| Week 1–4 | 15.1 | 10.7 | 0.19 | 71.4 | 71.9 | 0.42 |
| Week 5–8 | 28.6 | 13.3 | 0.06 | 76.2 | 76.7 | 0.22 |
| Week 9–12 | 35.7 | 20.0 | 0.05 | 80.0 | 78.8 | 0.37 |
| Week 1–12 | 27.8 | 16.2 | 0.12 | 75.9 | 74.8 | 0.32 |

The range of minimum and maximum values for percentage of days with no additional bronchodilator use at baseline was 0–88% in the fluticasone propionate (FP) group and 0–70% in the budesonide (BUD) group. The range for percentage of nights with no additional bronchodilator use at baseline was 0–88% in both groups. All other ranges were 0–100%.

bly related to study medication. The most common adverse events of any severity occurring during therapy are summarized in Table 6. There were no significant intergroup differences in frequency.

Mean serum cortisol levels, measured over the 12-week treatment period, increased by 12.2 nmol/l from baseline in patients treated with FP compared with a decrease of −4.9 nmol/l from baseline on BUD. In patients who did not take prednisolone during the trial (91% of patients on FP; 90% on BUD) the difference between FP and BUD was more marked (an increase of 13.5 nmol/l for FP compared with a decrease of −8.0 nmol/l for BUD). Analysis of log-transformed values revealed a statistically significant difference between the two treatment groups favouring FP ($p = 0.0002$). The adjusted geometric mean ratio was 1.03 for FP and 0.93 for BUD ($p = 0.0002$).

**Table 6.** Summary of the most common adverse events on therapy from case record forms

| | FP 800 µg n (%) | BUD 1 600 µg n (%) |
|---|---|---|
| Patients | 256 | 262 |
| Patients with adverse events | 158 (61.7) | 161 (61.5) |
| Upper respiratory tract infection | 55 (21.5) | 65 (24.9) |
| Exacerbation of asthma and related events | 37 (14.5) | 46 (17.6) |
| Rhinitis/sinusitis | 29 (11.3) | 21 (8.0) |
| Musculoskeletal pain | 23 (9.0) | 13 (5.0) |
| Bronchitis | 20 (7.8) | 16 (6.1) |
| Cerebrovascular | 17 (6.6) | 15 (5.7) |
| Sore throat | 15 (5.9) | 11 (4.2) |

Most common is defined as experienced by ≥4% of patients in each treatment group. Some patients reported more than one adverse event. BUD = budesonide; FP = fluticasone propionate.

There was, however, a statistically significant interaction between the baseline serum cortisol values and treatments ($p = 0.0004$). Suppression of the HPA axis of clinical concern was seen in six patients (2.3%) on FP compared with eleven (4.2%) of those who received BUD. However, this difference did not attain statistical significance.

### Discussion

FP 800 µg daily was found to be more effective than BUD 1 600 µg daily for the treatment of moderate-to-severe asthma in adults, with the primary efficacy variable of morning PEF showing a statistically significant advantage for FP over BUD. During the 12 weeks of treatment, morning PEF increased by 20.9 l/min in patients treated with FP compared with only 12.4 l/min on BUD. For purposes of comparison, an improvement of 20.0 l/min on therapy is generally considered to be of clinical significance.[16] Analysis of evening PEF, diurnal variation and percentage predicted PEF, as well as $FEV_1$, FVC and PEF measured at the clinic, confirms these findings, with statistically significant differences in favour of FP for all parameters.

International asthma treatment guidelines recommending inhaled corticosteroids as first-line therapy are likely to lead to an increased use of these agents in asthma.[1,2] A stepwise approach, which increases the dose with increasing severity of symptoms is recommended, meaning that some patients with moderate asthma may receive inhaled corticosteroids at higher dosages than are currently given as maintenance. Once symptoms resolve and lung function improves, the dose is then typically reduced to the minimum required to maintain control. In practice, however, many physicians initiate therapy at a high dose, which is then reduced once symptoms have been adequately controlled to establish the optimum maintenance dose.

Inhaled corticosteroids such as BDP and BUD are generally well tolerated at maintenance doses. However, studies have shown that high doses of these agents are associated with an increased risk of systemic adverse effects, possibly including suppression of the HPA axis, osteoporosis and growth retardation.[4–6] Such findings clearly indicate the need for inhaled corticosteroids which combine high topical potency with higher safety margins for systemic adverse effects for the treatment of symptomatic patients who are currently taking high doses of these drugs. The results of this study indicate that FP, as predicted by its potent *in vitro* profile, may offer this advantage.

The improvements in lung function seen during treatment with FP in this trial are consistent with the results of previous studies which have shown FP to be at least as effective as BUD or BDP, even when administered at half the dosage.[12–17] FP therefore appears to be at least twice as potent as these older inhaled corticosteroids *in vivo*, confirming *in vitro* data.[7–11]

Secondary efficacy variables of day- and night-time symptom score, additional bronchodilator use and exacerbations of asthma showed similar improvements in both treatment groups. Again, these results are consistent with those of previous studies.[12–17] Both patient groups experienced an improvement in their asthma symptoms and required less additional bronchodilator therapy. Night-time symptom severity and bronchodilator use were particularly improved. This is of clinical significance as both are possible factors which have a major effect on patient quality-of-life, although this was not formally studied in this trial.

The overall incidence and type of adverse events reported on therapy were found to be comparable in both treatment groups and only a minority of those adverse events reported were actually considered to be related to therapy in any way.

The potential for the two treatments to cause systemic effects was evaluated by measurement of serum cortisol levels. FP produced an increase in mean morning serum cortisol levels, whilst treatment with BUD decreased serum cortisol levels. The clinical importance of this is uncertain. Clinically relevant suppression of the HPA axis was seen in only 2.3% of patients treated with FP compared with 4.2% of those who received BUD during this trial.

In summary, the results of this study show that FP 800 μg daily is more effective than BUD 1 600 μg daily for the treatment of adults with moderate-to-severe asthma. Superior improvements were seen in PEF with FP, even at half the dose of BUD. In contrast to BUD, FP at this relatively high dose had less effect on HPA axis function as measured by serum cortisol levels, indicating a superior efficacy:safety ratio. These findings support the use of FP in adult patients who require inhaled corticosteroids to further improve their asthma control.

### References

1. National Heart, Lung and Blood Institute. International consensus report on diagnosis and treatment of asthma. *Eur Respir J* 1992; **5**: 601–641.
2. British Thoracic Society. Guidelines for management of asthma. *Thorax* 1993; **48**(suppl): S1–S24.
3. Levy ML, Stevenson IC on behalf of a UK study group. A comparison of the efficacy of inhaled fluticasone propionate 2 mg daily and a reducing course of oral prednisolone in the treatment of acute exacerbations of asthma. *American Thoracic Society, Seattle* 1995; A276.
4. Geddes D. Inhaled corticosteroids: benefits and risks. *Thorax* 1992; **47**: 404–406.
5. Boe J, Skoogh BE. Is long-term treatment with inhaled steroids hazardous? *Eur Respir J* 1992; **5**: 1037–1039.
6. Brown PH, Blundell G, Greening AP, Crompton GK. Hypothalamo-pituitary-adrenal axis suppression in asthmatics inhaling high dose corticosteroids. *Respir Med* 1991; **85**: 501–510.
7. Phillips GH. Structure-activity relationships of topically active steroids: the selection of fluticasone propionate. *Respir Med* 1990; **84**(suppl A): 19–23.
8. Harding SM. The human pharmacology of fluticasone propionate. *Respir Med* 1990; **84** (suppl A): 25–29.
9. Umland SP, Nahrebna DK, Rasac S, *et al.* Effects of mometasone furoate and other glucocorticoids on cytokine production from cultured peripheral blood CD4+ T cells. *J Allergy Clin Immunol* 1996; **97**: A423.
10. Kita H, Hagen JB, Gleich GJ. Fluticasone propionate is the most potent glucocorticoid in the induction of apoptosis of eosinophils. In: *Acute and Chronic Inflammation in the Respiratory Tract. International Respiratory Forum.* Colwood Publishing, 1995.
11. Schroeder JT, Stellato C, Lichtenstein LM, Bickel CA, Schleimer RP. Regulation of human basophil histamine release and IL-4 secretion by glucocorticoids. In: *Acute and Chronic Inflammation in the Respiratory Tract. International Respiratory Forum.* Colwood Publishing, 1995.
12. Fabbri L, Burge PS, Croonenborgh L, *et al.* Comparison of fluticasone propionate with beclomethasone dipropionate in moderate-to-severe asthma treated for one year. *Thorax* 1993; **48**: 817–823.
13. Ayres JG, Bateman ED, Lundbäck B, *et al.* High dose fluticasone propionate, 1 mg daily, versus fluticasone propionate, 2 mg daily, or budesonide, 1.6 mg daily, in patients with chronic severe asthma. *Eur Respir J* 1995; **8**: 579–586.
14. Leblanc P, Mink S, Keistinen T, *et al.* A comparison of fluticasone propionate 200 μg/day with beclomethasone dipropionate 400 μg/day in adult asthma. *Allergy* 1994; **49**: 380–385.
15. Lundbäck B, Alexander M, Day J, *et al.* Evaluation of fluticasone propionate (500 μg day$^{-1}$) administered either as dry powder via a Diskhaler® inhaler or pressurized inhaler and compared with beclomethasone dipropionate (1000 μg day$^{-1}$) administered by pressurized inhaler. *Respir Med* 1993; **87**: 609–620.
16. Langdon CG, Casey LJ, UK study group. Fluticasone propionate and budesonide in adult asthmatics: a comparison using dry-powder inhaler devices. *Br J Clin Res* 1994; **5**: 85–99.
17. Langdon CG, Thompson J, on behalf of a UK study group. A multicentre study to compare the efficacy and safety of inhaled fluticasone propionate and budesonide via metered-dose inhalers in adults with mild-to-moderate asthma. *Br J Clin Res* 1994; **5**: 73–84.

**Received 28 August 1996; accepted 10 September 1996**

## References

[PDF_00711] Ayres J. G., Bateman E. D., Lundbäck B., Harris T. A. (1995). High dose fluticasone propionate, 1 mg daily, versus fluticasone propionate, 2 mg daily, or budesonide, 1.6 mg daily, in patients with chronic severe asthma. International Study Group.. Eur Respir J.

[PDF_00685] Boe J., Skoogh B. E. (1992). Is long-term treatment with inhaled steroids in adults hazardous?. Eur Respir J.

[PDF_00687] Brown P. H., Blundell G., Greening A. P., Crompton G. K. (1991). Hypothalamo-pituitary-adrenal axis suppression in asthmatics inhaling high dose corticosteroids.. Respir Med.

[PDF_00708] Fabbri L., Burge P. S., Croonenborgh L., Warlies F., Weeke B., Ciaccia A., Parker C. (1993). Comparison of fluticasone propionate with beclomethasone dipropionate in moderate to severe asthma treated for one year. International Study Group.. Thorax.

[PDF_00693] Harding S. M. (1990). The human pharmacology of fluticasone propionate.. Respir Med.

[PDF_00715] Leblanc P., Mink S., Keistinen T., Saarelainen P. A., Ringdal N., Payne S. L. (1994). A comparison of fluticasone propionate 200 micrograms/day with beclomethasone dipropionate 400 micrograms/day in adult asthma.. Allergy.

[PDF_00718] Lundback B., Alexander M., Day J., Hebert J., Holzer R., Van Uffelen R., Kesten S., Jones A. L. (1993). Evaluation of fluticasone propionate (500 micrograms day-1) administered either as dry powder via a Diskhaler inhaler or pressurized inhaler and compared with beclomethasone dipropionate (1000 micrograms day-1) administered by pressurized inhaler.. Respir Med.

[PDF_00690] Phillipps G. H. (1990). Structure-activity relationships of topically active steroids: the selection of fluticasone propionate.. Respir Med.

